# Evaluating the relationship between familial poverty, *Helicobacter pylori* seropositivity, and all-cause mortality in the general US population

**DOI:** 10.3389/fpubh.2025.1578257

**Published:** 2025-06-17

**Authors:** Chenyu Jiang, Luqi Zhu, Wenyuan Yang, Zhenjun Yu, Weiwei Yang, Xiaolong Jin, Yaojian Shao

**Affiliations:** ^1^Department of Geriatric, Taizhou Central Hospital (Taizhou University Hospital), Taizhou, Zhejiang, China; ^2^Department of Critical Care Medicine, Taizhou Hospital of Zhejiang Province Affiliated to Wenzhou Medical University, Taizhou, Zhejiang, China; ^3^Department of Gastroenterology, Taizhou Central Hospital (Taizhou University Hospital), Taizhou, Zhejiang, China

**Keywords:** family poverty to income ratio, socioeconomic status, *Helicobacter pylori*, mortality, NHANES

## Abstract

**Purpose:**

Socioeconomic inequality is closely related to the incidence of *Helicobacter pylori* (*H. pylori*) infection and mortality outcomes. Accordingly, this study was designed with the goal of exploring the relationship between familial poverty, *H. pylori* seropositivity, and all-cause mortality among adults in the United States.

**Methods:**

Data from National Health and Nutrition Examination Survey (1999–2000) was used to conduct analysis. Family poverty to income ratio (PIR) was applied to evaluate socioeconomic status. The interplay between *H. pylori* serostatus and PIR was evaluated through univariate and multivariable approaches. The relationship between PIR, *H. pylori* serostatus, and the incidence of all-cause mortality was further assessed through Cox regression analysis, restricted cubic spline, and survival analysis.

**Results:**

A total of 3,573 individuals were included in this study. PIR values were found to be negatively associated with *H. pylori* seropositivity incidence after adjusting for potential covariates. Smooth curve fitting suggested that the relationship between these two variables was largely linear. Subgroup analyses confirmed that PIR values were still closely associated with *H. pylori* seropositivity independently. Moreover, multivariate Cox regression analysis demonstrated that lower PIR was associated with an increase in all-cause mortality in both *H. pylori* seropositivity and seronegative group, whereas *H. pylori* serostatus showed no association with all-cause mortality. Additional analysis using smooth curve fitting indicated a nonlinear relationship between PIR and all-cause mortality. The survival analysis further indicated that individuals with higher PIR values exhibited lower mortality rates, regardless of *H. pylori* serostatus.

**Conclusion:**

The present analyses reveal an inverse association between PIR values and *H. pylori* seropositivity and all-cause mortality. The relationship between PIR and all-cause mortality was not affected by *H. pylori* seropositivity. *H. pylori* serostatus is not a major risk factor for all-cause mortality. However, additional studies will be essential to better clarify the clinical relevance of these findings and to elucidate the underlying findings.

## 1 Introduction

*Helicobacter pylori* is a Gram-negative bacterium that can infect the stomach of humans, contributing to the incidence of a range of severe gastrointestinal disorders including peptic ulcers, gastritis, and gastric cancer (1). The estimated global prevalence of *H. pylori* infections is 44.3% (2), and the incidence of these infections differs markedly between developing and developed nations, with substantially higher odds in the former, particularly in countries in Latin America and Africa (3, 4). Recent epidemiological studies have indicated the prevalence of *H. pylori* has been declining globally between 2011 and 2022, particularly in African and Europe region (3, 5–7). However, the prevalence has remained relatively stable in Middle East (8). These observed differences in infection odds were associated with economic and social conditions (9, 10).

Socioeconomic status (SES) refers to an individual or group’s position in society and is often measured based on their education, income, or occupation (11). Lower SES is generally associated with higher *H. pylori* infection odds as it typically coincides with poorer dietary health, less sanitary conditions, and a worse overall living environment, all of which are risk factors for such infection (8, 10, 12). The complex nature of SES is reflected by the many different indicators used to assess this parameter, each of which is subject to certain limitations (13). Compared to other SES measures, like education level and occupational stratifications, household income better reflects living standards and access to services. The poverty-to-income ratio (PIR) is one such index that can be computed by dividing household income by the poverty threshold, and it is widely used in many reports (14–16). For the present analysis, the PIR was selected for use when examining the association between SES and *H. pylori* infection status in a nationally representative population of adults in the United States based on data collected through the National Health and Nutrition Examination Survey (NHANES).

Many researchers have also explored the association between SES and mortality outcomes, revealing that lower SES tends to be associated with a shorter life expectancy (17). Low SES is also established as a major risk factor related to the development of cancer and premature cancer-related mortality throughout the world. The World Health Organization (WHO) classified *H. pylori* as a Group 1 carcinogen associated with a high risk of gastric cancer and consequent mortality (18). In addition to gastric cancer and ulcers, *H. pylori* infection has been claimed to be associated with nearly 90 different systemic conditions, including cardiovascular, including cardiovascular and metabolic diseases, and other metabolic diseases (19, 20). In light of the above considerations, the present analyses sought to clarify the relationship between PIR values, *H. pylori* serostatus, and all-cause mortality based on the NHANES dataset.

## 2 Materials and methods

### 2.1 Data source and study population

The cross-sectional NHANES study was a large, nationally representative survey implemented by the National Center for Health Statistics (NCHS) with the goal of assessing the nutritional and health status of adults in the United States. The Ethics Review Committee of the NCHS approved this study, and all participants gave informed written consent to participate. For the present study, adults 20 + years of age with *H. pylori* IgG levels information from the NHANES 1999–2000 cycle who participated in subsequent survey cycles were included. Participants lacking PIR data, education level information, or *H. pylori* IgG levels were also excluded from these analyses.

**Figure 1:**
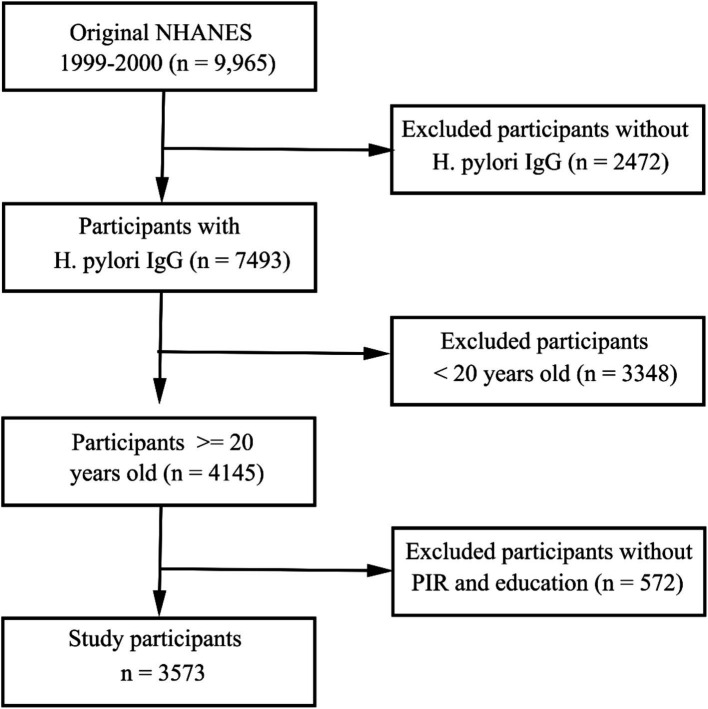
Flow chart overview of the selection of eligible patients from the NHANES 1999–2000 cohort.

The 1999–2000 NHANES cycle included 9,965 subjects of whom 3,753 were found to meet the established inclusion criteria (Figure 1). The analyses conducted with the available data were representative of the population, so we did not deal with missing values in the covariates.

### 2.2 Socioeconomic status analyses

PIR values were selected as the primary index when evaluating SES in the present study. PIR values are based upon the poverty guidelines released by the Department of Health and Human Services, taking into consideration both household size and the relevant poverty threshold of the appropriate year and state when assessing the SES of a given individual to provide a more precise reflection of actual poverty status. PIR is adjusted for the size and composition of the household, and the age of family members, and is updated annually for inflation. PIR = 1 means the income equals the federal poverty level. To ensure adequate participant representation in each category for analytical purposes, the PIR was stratified into three levels based on tertiles. Education level was regarded as another important covariate for SES analyses, with participants being classified into those with an education level of less than high school, high school or equivalent, or college and above.

### 2.3 *Helicobacter pylori* serostatus

Human serum samples were analyzed for *H. pylori-*specific IgG via ELISA (Wampole Laboratories) (21). These ELISAs offer similar sensitivity, specificity, and repeatability to those afforded by other serological assays based on hemagglutination, complement fixation, immunofluorescence, or other factors (22–25). Serum IgG antibody levels below 0.9 were considered negative, while any values above this threshold were considered seropositive for *H. pylori* as in prior reports (23, 26). Note that seropositivity encompasses both current and past infections. The serologic methods exhibited a sensitivity above 90% and a specificity of around 80% (27–29). Although *H pylori* serology has lower specificity compared to other noninvasive tests, its superior sensitivity and low-cost make it a suitable noninvasive test for ruling out *H pylori* infection (30–32). Specific measurement methods and validity can be found at https://wwwn.cdc.gov/Nchs/Nhanes/1999-2000/LAB11.htm#LBXHP1.

### 2.4 Participant follow-up

Records for study participants were linked to the National Death Index files^1^ to assess patient mortality outcomes. Participants were followed from the baseline interview until death or December 31, 2019. The median follow-up durations for *H. pylori* seropositivity and seronegative patients were 233 (IQR: 177, 242) and 234 (IQR: 224, 241) months, respectively.

### 2.5 Other covariates

Covariates with the potential to impact *H. pylori* serostatus were taken into consideration when conducting these analyses. These included demographic variables such as age (years), gender (male/female), and race/ethnicity (Mexican American, Other Hispanic, Non-Hispanic White, Non-Hispanic Black, Other race/ethnicity). Relevant lifestyle variables included smoking status (never [<100 cigarettes lifetime], former [>100 cigarettes lifetime and smoke not at all now], now [>100 cigarettes lifetime and smoke some days or everyday]) and drinking status (never [<12 lifetime drinks], former [12 + drinks per year but none in the last year], current light/moderate drinker [≤1 or ≤2 daily drinks for females and males, respectively, over the past 12 months], current heavy drinker [>1 or >2 daily drinks for females and males, respectively, over the past 12 months]). Health-related variables included body mass index (BMI; normal [< 25 kg/m^2^], overweight [25–30 kg/m^2^], obese [≥30 kg/m^2^]), diabetes, and hypertension. Individuals were considered hypertensive if they were using antihypertensive drugs, self-reported a hypertension diagnosis, or exhibited systolic (diastolic) blood pressure values ≥140 mm Hg (≥90 mm Hg). Individuals were considered to have diabetes if they self-reported a diagnosis of diabetes, were using insulin or any other antidiabetic medications, exhibited a fasting glucose ≥ 126 mg/dL, an HbA1c ≥ 6.5%, or a serum glucose level 2 h after a 75 g glucose load (OGTT) ≥ 200 mg/dL. Analyzed laboratory parameters included triglycerides (mg/dL), total cholesterol (mg/dL), uric acid levels (mg/dL), albumin levels (g/dL), creatinine levels (mg/dL), and blood urea nitrogen (mg/dL).

### 2.6 Statistical analyses

To account for the complex multi-stage cluster design of the NHANES study, appropriate sample weight values were employed to ensure that the adjusted data reflects the characteristics of the U. S. population. The baseline characteristics of the participants by *H. pylori* serologic status classifications were compared using chi-square tests or t-tests. Categorical variables were reported as numbers (weighted percentage) and analyzed using chi-square tests. Continuous variables were reported as means ± standard error (SE) and analyzed using t-tests.

Associations between PIR values and *H. pylori* seropositivity were assessed through survey-weighted univariate and multivariable logistic regression analyses that were used to compute odds ratios (ORs) and 95% confidence intervals (CIs). Three different models were used to conduct these analyses. In Model 1, education, another SES factor were adjusted. In Model 2, the demographic characteristics of the general population, including age, gender, race/ethnicity, and BMI were additionally adjusted. Considering the potential impact of chronic underlying diseases and living conditions, smoking, alcohol intake, hypertension, diabetes mellitus, and albumin levels (g/dL) were further adjusted in Model 3. The potential non-linearity of the association between PIR and *H. pylori* serostatus was assessed using restricted cubic spline (RCS) analyses adjusted for relevant confounding variables. Associations between these variables were also assessed in different demographic subgroups. Relationship between PIR values, *H. pylori* serostatus, and all-cause mortality were assessed through univariate and multivariable Cox regression analyses. To optimize data use and reduce overadjustment for mediating variables, two models were estimated: in model 1, the demographic characteristics including age, gender, education, race/ethnicity, and BMI were adjusted; in model 2, chronic underlying diseases and living conditions including hypertension, and diabetes mellitus were further adjusted. PIR was further transformed into tertiles and used as a categorical variable to conduct survival analyses using Kaplan–Meier curves and log-rank tests evaluating event-free survival rates as a function of PIR level and *H. pylori* seropositivity. R (v 4.2.2) was used to conduct all analyses, with a two-sided *p* < 0.05 as the threshold used to define significance.

## 3 Results

### 3.1 Participant characteristics

This study ultimately enrolled 3,573 subjects (weighted n = 161,312,594). Main characteristics and laboratory parameters for these participants stratified according to *H. pylori* serostatus are presented in Table 1. In total, 45.0% (weighted percentage = 31.01%) of this study cohort exhibited *H. pylori* seropositivity, and seropositive individuals were more likely to be older, current smokers, former drinkers, individuals with a lower education level, individuals with a lower PIR and individuals with hypertension, diabetes, and lower albumin levels. There was no association found between *H. pylori* serostatus and gender, BMI, total cholesterol, triglyceride, creatinine, uric acid, or blood urea nitrogen level.

**Table 1:** Baseline weighted participant characteristics and laboratory parameters grouped according to *Helicobacter pylori* serostatus.

Variable	All participants	HP seronegative	HP seropositive	*p*-value
No. of participants	3,573	1965 (68.99%)	1,608 (31.01%)	
Age (years)	44.48 (0.38)	42.76 (0.48)	48.32 (0.74)	< 0.0001
Fast triglyceride (mg/dl)	143.71 (4.12)	140.14 (6.08)	152.23 (6.19)	0.24
Fast total cholesterol (mg/dl)	202.66 (1.23)	202.67 (1.65)	202.64 (2.15)	0.99
Albumin (g/dl)	4.49 (0.02)	4.51 (0.02)	4.44 (0.02)	< 0.001
Uric acid (mg/dl)	5.30 (0.04)	5.27 (0.04)	5.37 (0.06)	0.2
Urea nitrogen (mg/dl)	14.08 (0.11)	14.01 (0.12)	14.24 (0.20)	0.28
Creatinine (mg/dl)	0.75 (0.01)	0.75 (0.01)	0.75 (0.01)	0.72
PIR	2.92 (0.12)	3.16 (0.13)	2.39 (0.11)	< 0.0001
Sex				0.59
Female	1880 (51.72%)	1,064 (69.31%)	816 (30.69%)	
Male	1,693 (48.28%)	901 (68.65%)	792 (31.35%)	
Race/ethnicity				< 0.0001
Mexican American	951 (6.04%)	306 (37.17%)	645 (62.83%)	
Non-Hispanic Black	635 (9.85%)	269 (47.69%)	366 (52.31%)	
Non-Hispanic White	1,644 (71.43%)	1,236 (78.39%)	408 (21.61%)	
Other Hispanic	228 (8.30%)	91 (43.46%)	137 (56.54%)	
Other Race/ethnicity	115 (4.38%)	63 (55.92%)	52 (44.08%)	
Educational status				< 0.0001
Less than high school	633 (6.63%)	156 (27.67%)	477 (72.33%)	
High school or equivalent	1,494 (41.50%)	769 (62.02%)	725 (37.98%)	
College or above	1,446 (51.87%)	1,040 (79.85%)	406 (20.15%)	
BMI (kg/m2)	28.02 (0.22)	27.99 (0.28%)	28.10 (0.16)	0.68
<25	1,111 (35.32%)	656 (70.10%)	455 (29.90%)	
25–30	1,264 (34.00%)	672 (68.99%)	592 (31.01%)	
>30	1,159 (29.83%)	618 (67.57%)	541 (32.43%)	
Missing	39 (0.86%)	19 (72.85%)	20 (27.15%)	
DM				< 0.001
No	2,911 (89.56%)	1,644 (69.91%)	1,267 (30.09%)	
Yes	452 (8.02%)	178 (54.82%)	274 (45.18%)	
Missing	210 (2.42%)	143 (82.13%)	67 (17.87%)	
Hypertension				0.01
No	2,129 (67.60%)	1,250 (71.32%)	879 (28.68%)	
Yes	1,444 (32.40%)	715 (64.13%)	729 (35.87%)	
Coffee intake				0.18
No	1702 (49.19%)	964 (70.86%)	738 (29.14%)	
Yes	1752 (47.78%)	940 (67.19%)	812 (32.81%)	
Missing	119 (3.03%)	61 (67.08%)	58 (32.92%)	
Smoke				0.01
Never	1895 (51.45%)	1,073 (72.19%)	822 (27.81%)	
Former	944 (24.05%)	514 (69.08%)	430 (30.92%)	
Now	728 (24.40%)	375 (62.23%)	353 (37.77%)	
Missing	6 (0.10%)	3 (51.29%)	3 (48.71%)	
Alcohol drinking				< 0.001
Never	512 (11.28%)	262 (65.17%)	250 (34.83%)	
Former	707 (15.91%)	339 (60.44%)	368 (39.56%)	
Moderate	466 (16.10%)	293 (73.23%)	173 (26.77%)	
Mild	1,065 (32.21%)	656 (74.14%)	409 (25.86%)	
Heavy	649 (20.30%)	344 (68.77%)	305 (31.23%)	
Missing	174 (4.21%)	71 (56.99%)	103 (43.01%)	

Hp, *Helicobacter pylori*; PIR, family income-to-poverty ratio; BMI, body mass index; DM, diabetes mellitus; SE, standard error [Continuous variables were showed as means (SE), and categorical variables were reported as numbers (weighted percentage)].

### 3.2 Relationships between PIR levels and *Helicobacter pylori* seropositivity

**Figure 2:**
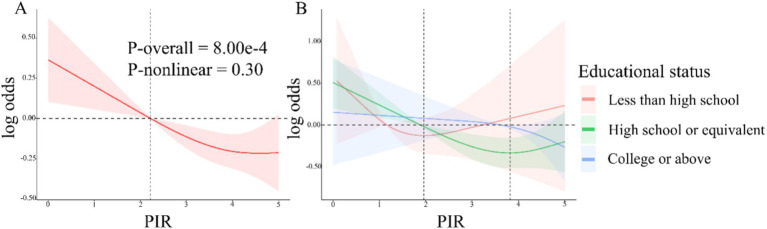
**(A)** RCS analyses of the association between PIR values and *Helicobacter pylori* serostatus under model 3. Red shaded area represent 95% confidence intervals. **(B)** Associations between PIR levels and *H. pylori* serostatus stratified according to educational status. The shaded areas in orange, green and blue represent the confidence intervals for less than high school, high school or equivalent, and college or above educational status.

*H. pylori* seropositivity was not meaningfully associated with gender, BMI, or coffee consumption, but was strongly associated with decreasing values of PIR (OR 0.74 [95% CI: 0.70, 0.79], Table 2) and very strongly associated with lower levels of educational status (HS or equivalent OR 0.23 [95% CI: 0.14, 0.34]; College or above OR 0.10 [0.06, 0.15]). In multivariable logistic regression analyses of the association between PIR and *H. pylori* serostatus (Table 3), a crude unadjusted model revealed that elevated PIR levels were associated with the decline in the odds of *H. pylori* seropositivity (OR = 0.74 95% CI: (0.70, 0.79), *p* < 0.0001). This inverse association persisted after adjustment for education in Model 1 (OR = 0.83, 95% CI: (0.79, 0.88), *p* < 0.0001) and adjustment for education, age, gender, race/ethnicity, and BMI in Model 2 (OR = 0.88, 95% CI: (0.82, 0.95), *p* < 0.01). Under Model 3, the corresponding OR was 0.90 (95% CI, 0.85–0.95), such that each unit increase in PIR values was associated with a 10% reduction in the odds of *H. pylori* seropositivity. When assessing PIR tertiles, in the crude model, participants in the two higher PIR tertiles (T2/3) had higher odds of *H. pylori* seropositivity (T2: OR = 0.63, 95% CI, 0.47–0.84; T3: OR = 0.33, 95% CI, 0.25–0.43) as compared to patients in the lowest tertile (T1). This relationship remained intact after adjusting for educational status in Model 1 and additionally adjusting for age, gender, race/ethnicity, and BMI in Model 2. There was no statistical difference in T2 after further adjustment for smoking, alcohol intake, hypertension, diabetes mellitus, and albumin levels (g/dL) in Model 3, whereas, the ORs of T3 were 0.69 (95%CI, 0.56–0.85, P for tend = 0.001). Smooth curve fitting analyses adjusted for relevant covariates demonstrated that the inverse association between PIR levels and *H. pylori* serostatus was largely linear (P-overall = 8.00 × 10^−4^ and P-nonlinear = 0.30). In the educational status stratification, a negative non-linear association was noted between PIR level and *H. pylori* seropositivity for subjects with college or higher levels of education. The RCS analyses revealed U-shaped curves for less than high school and high school or equivalent education levels subjects, with inflection points at 1.96 and 3.82, respectively (Figure 2). RCS analyses performed when stratifying subjects according to gender, BMI, hypertension, and diabetes status are presented in Supplementary Figure 1.

**Table 2:** Sample-weighted univariate analysis of the factors associated with the odds of *H. pylori* seropositivity in the national health and nutrition examination survey 1999–2000 cohort.

Variable	OR	95% CI	*p* value
Age (years)	1.02	1.01–1.03	<0.0001
Fast triglyceride (mg/dl)	1	1.00–1.00	0.25
Fast total cholesterol (mg/dl)	1	1.00–1.00	0.99
Albumin (g/dl)	0.52	0.40–0.67	<0.0001
Uric acid (mg/dl)	1.04	0.97–1.12	0.20
Urea nitrogen (mg/dl)	1.01	0.99–1.03	0.27
Creatinine (mg/dl)	0.97	0.84–1.13	0.70
PIR	0.74	0.70–0.79	<0.0001
Sex			
Female	reference		reference
Male	1.03	0.91–1.16	0.59
Race/ethnicity			
Mexican American	reference		reference
Non-Hispanic Black	0.65	0.44–0.95	0.03
Non-Hispanic White	0.16	0.12–0.23	<0.0001
Other Hispanic	0.77	0.50–1.19	0.21
Other Race/ethnicity	0.47	0.27–0.80	0.01
Educational status			
College or above	reference		reference
Less than high school	10.36	6.46–16.63	<0.0001
High school or equivalent	2.43	1.90–3.10	<0.0001
BMI (kg/m2)	1	0.99–1.02	0.69
<25	reference		reference
25–30	1.05	0.84–1.32	0.63
>30	1.12	0.88–1.44	0.32
DM			
No	reference		reference
Yes	1.91	1.30–2.82	0.003
Hypertension			
No	reference		reference
Yes	1.39	1.11–1.75	0.01
Coffee intake			
No	reference		reference
Yes	1.19	0.94–1.50	0.14
Smoke			
Never	reference		reference
Former	1.16	0.88–1.54	0.27
Now	1.58	1.22–2.03	0.002
Alcohol drinking			
Never	reference		reference
Former	1.22	0.83–1.80	0.27
Moderate	0.68	0.49–0.96	0.03
Mild	0.65	0.48–0.88	0.01
Heavy	0.85	0.51–1.41	0.49

Hp, *Helicobacter pylori*; PIR, family income-to-poverty ratio; BMI, body mass index; DM, diabetes mellitus.

**Table 3:** Sample-weighted multivariable logistic regression analyses of the relationships between poverty-to-income ratio values and *H.* seropositivity.

Variable	Crude model			Model 1			Model 2			Model 3		
	OR	95%CI	*p* value	OR	95%CI	*p* value	OR	95%CI	*p* value	OR	95%CI	*p* value
PIR	0.74	0.70–0.79	<0.0001	0.83	0.79–0.88	<0.0001	0.88	0.82–0.95	0.01	0.9	0.85–0.95	<0.001
PIR tertile												
T1	reference			reference			reference			reference		
T2	0.63	0.47–0.84	0.005	0.77	0.60–0.99	0.04	0.83	0.56–1.24	0.24	0.86	0.64–1.15	0.27
T3	0.33	0.25–0.43	<0.0001	0.52	0.42–0.65	<0.0001	0.65	0.48–0.88	0.02	0.69	0.56–0.85	0.002
*P* for trend			< 0.001			< 0.001			0.01			0.001

Model 1: The educational status was adjusted. Model 2: The adjusted for educational status, age, gender, race/ethnicity, BMI (kg/m2) were adjusted. Model 3: The educational status, age, gender, race/ethnicity, BMI (kg/m2), smoking, alcohol intake, hypertension, diabetes mellitus, albumin levels (g/dL) were adjusted. Hp, *Helicobacter pylori*; PIR, family income-to-poverty ratio; BMI, body mass index; DM, diabetes mellitus.

### 3.3 Subgroup analysis

The robustness of the above results was next evaluated through a series of subgroup analyses (Table 4). This approach revealed associations between PIR values and *H. pylori* seropositivity among subjects with high school or equivalent (OR = 0.78, 95% CI, 0.71–0.86) and college or above (OR = 0.86, 95% CI, 0.77–0.95) educational status relative to other subgroups. These associations were similarly noted for participants of Mexican American (OR = 0.86, 95% CI, 0.75–0.99), Non-Hispanic Black (OR = 0.87, 95% CI, 0.79–0.96), non-Hispanic White (OR = 0.78, 95% CI, 0.71–0.86), and other race/ethnicity (OR = 0.79, 95% CI, 0.64–0.97), whereas these associations were not detected in individuals of other Hispanic race/ethnicity or participants with less than a high school education. No significant interactions between PIR and *H. pylori* were detected in these subgroup analyses (P for interaction > 0.05).

**Table 4:** Sample-weighted subgroup analyses of the association between poverty-to-income ratio values and *H. pylori* seropositivity.

Variable	OR	95% CI	*p* value	*P* for interaction
Sex				0.4
Female	0.72	0.67–0.78	<0.0001	
Male	0.76	0.69–0.84	<0.0001	
Race/ethnicity				0.11
Mexican American	0.86	0.75–0.99	0.04	
Non-Hispanic Black	0.87	0.79–0.96	0.01	
Non-Hispanic White	0.78	0.71–0.86	<0.0001	
Other Hispanic	1.06	0.71–1.60	0.73	
Other Race/ethnicity	0.79	0.64–0.97	0.03	
Educational status				0.07
Less than high school	1.16	0.82–1.65	0.37	
High school or equivalent	0.78	0.71–0.86	<0.0001	
College or above	0.86	0.77–0.95	0.01	
BMI (kg.m2)				0.08
<25	0.78	0.71–0.87	<0.001	
25–30	0.69	0.63–0.76	<0.0001	
>30	0.75	0.69–0.82	<0.0001	
DM				0.08
No	0.76	0.70–0.81	<0.0001	
Yes	0.66	0.57–0.76	<0.0001	
Hypertension				0.7
No	0.74	0.68–0.79	<0.0001	
Yes	0.76	0.67–0.86	<0.001	
Coffee intake				0.71
No	0.75	0.66–0.84	<0.001	
Yes	0.73	0.67–0.79	<0.0001	
Smoke				0.39
Never	0.73	0.65–0.82	<0.0001	
Former	0.73	0.65–0.82	<0.0001	
Now	0.81	0.71–0.91	0.002	
Alcohol drinking				0.57
Never	0.72	0.58–0.89	0.01	
Former	0.69	0.58–0.83	<0.001	
Moderate	0.74	0.61–0.90	0.01	
Mild	0.73	0.66–0.81	<0.0001	
Heavy	0.83	0.70–0.98	0.03	

Hp, *Helicobacter pylori*; PIR, family income-to-poverty ratio; BMI, body mass index; DM, diabetes mellitus.

### 3.4 Association with all-cause mortality

**Figure 3:**
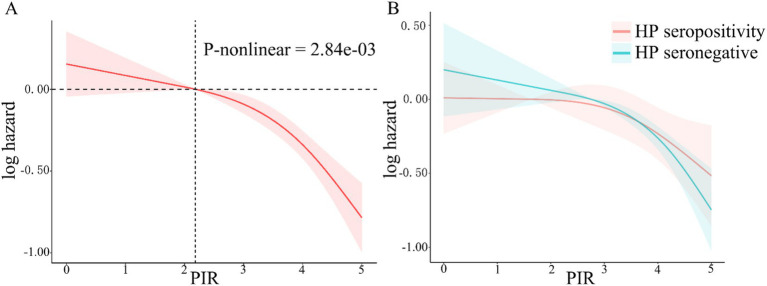
RCS analyses of the relationship between PIR values and all-cause mortality risk in total participant (**A**, the red shaded area represent 95% confidence intervals) and stratified according to *H. pylori* serostatus (**B**, the red and green shaded area, respectively, represent 95% confidence intervals of *H. pylori* seropositivity and seronegative).

**Figure 4:**
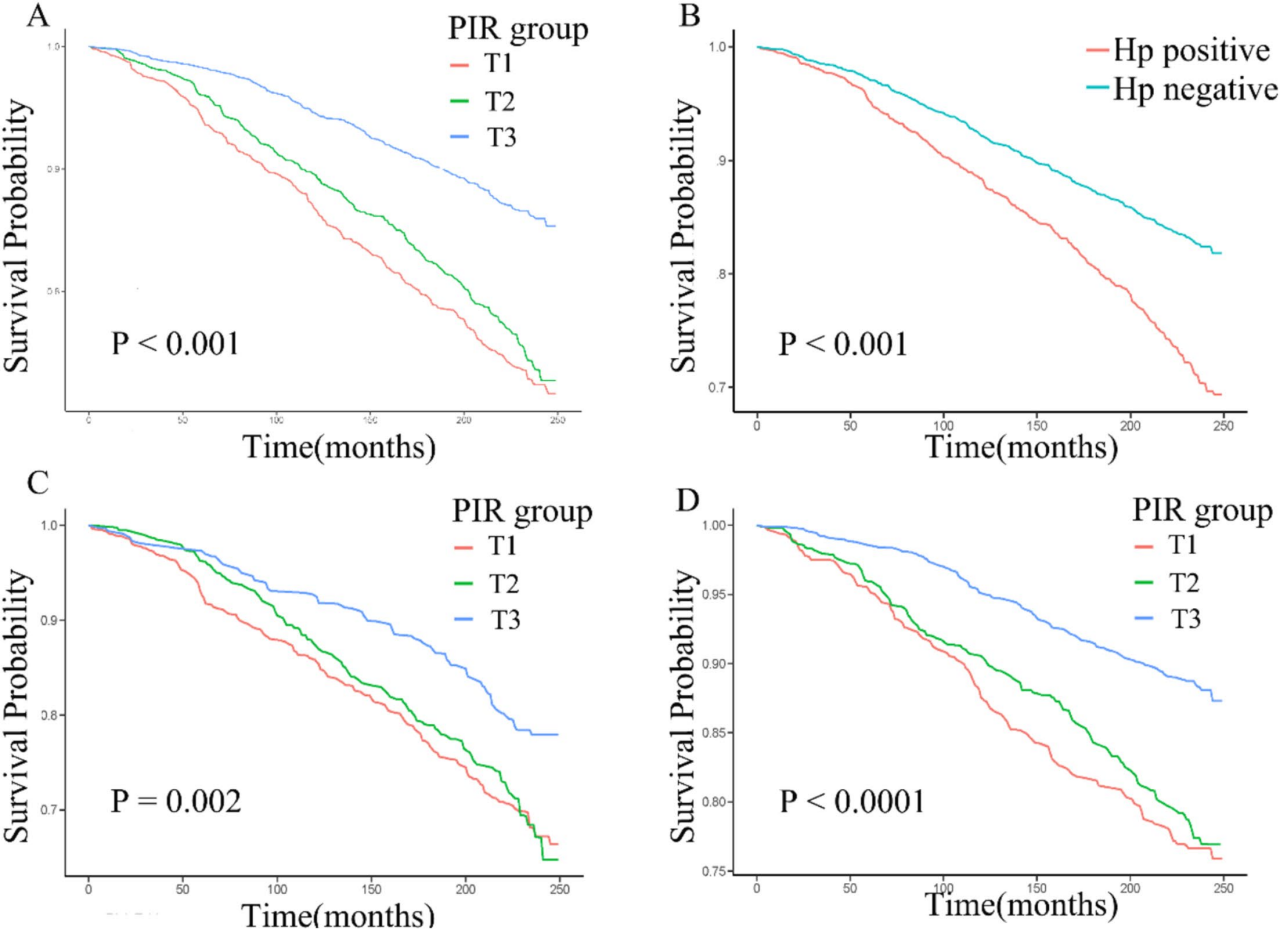
**(A–D)** Kaplan–Meier curves corresponding to different PIR tertiles in the overall cohort **(A)**, *H. pylori* seropositivity groups in the overall cohort **(B)**, PIR tertiles in *H. pylori* seropositivity individuals **(C)**, and PIR tertiles in *H. pylori* seronegative individuals **(D)**.

During the median 243 months of follow-up, 1,104 (31%, weighted percentage = 21.08%) deaths were recorded and one subject was lost to follow-up. As continuous variable, in Model 2, for each unit increase in the PIR, adjusted hazard ratio (HR) for all-cause mortality was 0.87 (95% CI: 0.77–0.99) in *H. pylori* seropositivity participants and 0.81 (95% CI: 0.74–0.88) in *H. pylori* seronegative population (Table 5). When assessing PIR as categorical variable, after adjusting age, gender, education, race/ethnicity, BMI, hypertension, and diabetes mellitus, compared to the T1 group, both *H. pylori* seropositivity and seronegative participants in the T3 group exhibited a lower risk of death (respectively, *H. pylori* seropositivity HR: 0.58, 95% CI: 0.35–0.95; *p* = 0.04; *H. pylori* seronegative HR: 0.45, 95% CI: 0.31–0.65; *p* < 0.001; Table 5). The subgroup analyses showed that the relationship between PIR and all-cause mortality remained consistent across all subgroups (Supplementary Table 1). Coffee intake and smoke modified the relationship between PIR and all-cause mortality (P for interaction < 0.05). In crude model, *H. pylori* seropositivity was associated with an increased risk of all-cause mortality (Table 6). However, there was no association observed between *H. pylori* serostatus and all-cause mortality after adjusting for correlated covariates in Model 1 and Model 2. Based on the subgroup analyses, we found that the associations of *H. pylori* serostatus and all-cause mortality was still existed in some subsets of the population (Supplementary Table 2). The relationship between *H. pylori* serostatus and all-cause mortality was influenced by sex (P for interaction < 0.001). RCS curves indicated the nonlinear relations between PIR values and all-cause mortality risk in this cohort (P-nonlinear = 2.84 × 10^−3^ Figure 3A). In the *H. pylori* serostatus subgroup, a negative relationship was observed between PIR and all-cause mortality in *H. pylori* seropositivity and seronegative group (Figure 3B). When regarding PIR values as a categorical variable, Kaplan–Meier curves revealed that both lower PIR values and *H. pylori* seropositivity were linked to an increase in all-cause mortality risk (Figures 4A,B). Relative to PIR tertiles 1 and 2, participants in tertile 3 presented with a lower risk of mortality irrespective of *H. pylori* seropositivity (Figures 4C,D).

**Table 5:** Sample-weighted multivariable Cox regression analyses of the relationships between poverty-to-income ratio values and all-cause mortality stratified according to *H. pylori* serostatus.

Variable	Crude model			Model 1			Model 2		
	HR	95%CI	*p* value	HR	95%CI	*p* value	HR	95%CI	*p* value
HP seropositivity									
PIR	0.9	0.83–0.97	0.01	0.86	0.76–0.97	0.02	0.87	0.77–0.99	0.03
PIR tertile									
T1	reference			reference			reference		
T2	1	0.78–1.28	1	0.9	0.69–1.16	0.41	0.93	0.70–1.23	0.6
T3	0.63	0.44–0.88	0.01	0.57	0.36–0.90	0.02	0.58	0.35–0.95	0.03
*P* for trend			0.01			0.02			0.04
HP seronegative									
PIR	0.82	0.78–0.87	<0.0001	0.81	0.74–0.89	<0.0001	0.81	0.74–0.88	<0.0001
PIR tertile									
T1	reference			reference			reference		
T2	0.93	0.69–1.28	0.66	0.78	0.60–1.01	0.06	0.78	0.58–1.03	0.08
T3	0.46	0.35–0.61	<0.0001	0.46	0.32–0.65	<0.0001	0.45	0.31–0.65	<0.001
*P* for trend			< 0.001			<0.0001			<0.0001

Model 1: The adjusted for educational status, age, gender, race/ethnicity, BMI (kg/m2) were adjusted. Model 2: The educational status, age, gender, race/ethnicity, BMI (kg/m2), hypertension, and diabetes mellitus were adjusted. Hp, *Helicobacter pylori*; PIR, family income-to-poverty ratio; BMI, body mass index.

**Table 6:** Sample-weighted multivariable Cox regression analyses of the relationships between *H. pylori* serostatus and all-cause mortality.

Variable	Crude model			Model 1			Model 2		
	HR	95%CI	*p* value	HR	95%CI	*p* value	HR	95%CI	*p* value
HP antibody levels	1.27	1.15–1.39	<0.0001	1.01	0.94–1.08	0.82	1.01	0.95–1.08	0.70
HP seronegative	reference			reference			reference		
HP seropositive	1.78	1.41–2.25	<0.0001	1.02	0.87–1.20	0.82	1.03	0.88–1.20	0.74

Hp, *Helicobacter pylori*.

## 4 Discussion

In the present analyses, the associations between SES, *H. pylori* seropositivity, and all-cause mortality were assessed based on data from individuals included in the NHANES 1999–2000 cohort. PIR values and *H. pylori* seropositivity were applied as proxy variables for SES and *H. pylori* seropositivity. Overall, rising PIR levels were associated with a decline in *H. pylori* seropositivity. In subgroup analyses, this relationship remained stable across demographic groups. PIR was also associated with participant mortality irrespective of *H. pylori* seropositivity status. There is an association between *H. pylori* serostatus and all-cause mortality; however, this association is affected by numerous confounding factors.

An estimated 31.01% (weighted percentage) of the participant population in this study was infected with *H. pylori.* The proportion for *H. pylori* seropositivity in this study is similar to another study, which found that the seropositivity rate of *H. pylori* among general US adult population was 30.7% (33). The seroprevalence of *H. pylori* exhibited variations across different age groups, with a reported prevalence of 26.4% among US children aged 6–16 (34). During the same period, the highest prevalence was reported in Eastern Mediterranean region (82.7%), and the lowest in European region (46.2%) (7). While odds of such infections have been declining markedly in industrialized Western nations in recent years, they remain particularly high in developing nations (4, 35). In addition, our findings demonstrate racial and ethnic disparities in HP seroprevalence, with higher rates among minority groups, consistent with results from related studies (36, 37). The observed variations in *H pylori* prevalence across different age groups, race/ethnicity, and geography were associated with SES (38, 39). In Japan, living in crowded environments with many siblings was reported to be associated with a greater risk of *H. pylori* infection in younger individuals (10). This suggests that this bacterium can be transmitted within families such that children in impoverished and crowded areas face particularly elevated odds of infection owing to poorer hygienic conditions (40, 41).

Epidemiological research has shown that higher household income levels are associated with the adoption of healthier but more costly lifestyles at an early point in life (42). Those from lower-income household groups are more frequently exposed to infections due to factors including a lack of quality health service access, overcrowding and poor neighborhood environments (43, 44). Previous research has demonstrated that the lowest SES group suffered from the highest mean pathogen burden (45, 46). Once infection has been established, these individuals may lack the ability to control it (47). Our findings indicated decreased PIR was associated with *H. pylori* seropositivity. It is important to acknowledge the limitations of PIR values, including variations in income and wealth among different regions, as well as the potential influence of income instability on PIR fluctuations. Additionally, the PIR percentile classification may not reflect roughly homogenous standards of living within each category, particularly in comparison to the categorization scheme recommended in the NHANES Guidelines (48). This disparity in classification methods could introduce bias in our findings, as individuals may be misclassified based on differing categorization criteria. Nevertheless, PIR serves as a reasonably objective indicator of socioeconomic status and is commonly employed in investigating associations with chronic illnesses (14, 49, 50). Educational status is another SES indicator that is closely associated with measures of personal hygiene-related factors. In this study, *H. pylori* seropositivity odds were consistently found to be higher among individuals with lower educational status, whereas they were markedly lower among individuals with a college education or higher. Higher level of education are associated with improved knowledge of sanitation and approaches to eliminating unsanitary conditions, which may account for the reduced odds of *H. pylori* seropositivity in individuals with higher educational attainment (17, 51). The present results align well with prior evidence indicating that PIR values are inversely associated with *H. pylori* seropositivity (7, 34, 51). Smooth curve fitting and RCS approaches also confirmed that the relationship between PIR values and *H. pylori* serostatus was largely linear. Subgroup analyses additionally revealed that this association was maintained in all analyzed demographic groups with no significant interactions. PIR was independently associated with *H. pylori* seropositivity, and Cox regression analysis and survival analyses additionally revealed an increase in all-cause mortality risk with decreasing PIR values. These data are consistent with past reports identifying lower socioeconomic status as a major risk factor associated with the incidence of premature mortality (52–54). The disproportionate burden of persistent infection among disadvantaged groups may be an important determinant of disparities in long-term health (55). A substantial cumulative burden of chronic infections has the potential to induce an elevated state of inflammation and accelerate the onset of chronic ailments and mortality (34). Among the major pathogens, *H. pylori* is an important contributor to infection-related cancer deaths (53, 54, 56) and cardiovascular disease (57). In the present study, the result of crude model and Kaplan–Meier curve indicated that *H. pylori* seropositivity were associated with higher all-cause mortality (Figure 4B). However, after adjusting for covariates, *H. pylori* seropositivity was not associated with all-cause mortality. The result showed that the association *H. pylori* seropositivity and all-cause mortality was influenced by potential covariates. Previous evidence suggests that while *H. pylori* has a complex role in human health, it is not a major risk factor for all-cause mortality (58). The RCS curve showed a similar relationship between PIR and all-cause mortality in *H. pylori* seropositivity and seronegative groups. The result of weighted multivariable Cox regression analyses also demonstrated that PIR was negatively associated with all-cause mortality, regardless of *H. pylori* serostatus. The findings show that *H. pylori* seropositivity affects health, but it was not linked to all-cause mortality, which aligns with previous studies. Serology is common for general population assessment of *H. pylori* status. However, it is important to acknowledge the inherent limitations of this diagnostic test, as *H. pylori* serologic status can not differentiate a current or past infection. In addition, the varying availability of healthcare services among different SES populations during the follow-up survey may contribute to the disparities in *H. pylori* eradication rates. These limitations might potentially influence the result of the analysis. The findings of a meta-analysis suggest that *H. pylori* eradication reduced gastric cancer-related mortality but did not appear to affect all-cause mortality (59).

The NHANES survey compiles a range of health- and nutrition-related parameters from a nationally representative population of individuals from the United States, making it an invaluable resource for hypothesis generation and the assessment of relationships between particular exposures and health-related outcomes. In addition, this study is subject to certain limitations. First, both PIR values and Hp serological status were obtained at baseline; therefore, a causal relationship between the PIR values and Hp serological status cannot be established. Second, we employed *H. pylori* serological data for detecting *H. pylori* infection. It’s important to note that while the sensitivity of serological test is above 90%, its specificity is only about 80%. This means there’s a possibility of misclassification in this study. For example, patients with prior infections may test positive in the serological test. Third, the association between PIR and *H. pylori* seropositivity could be influenced by unmeasured or residual confounding factors and misclassification of *H. pylori* status and other variables. Fourth, consecutive PIR and *H. pylori* status changes during follow-up were not recorded and the majority of deaths are unrelated to *H. pylori* infection. These factors could lead to opaque recommendations or misinterpretations in the analysis of all-cause mortality. Fifth, our research conclusion only applies to Americans, and may not be generalizable to other regions. Sixth, weighted analysis may dilute the observations in oversampled minority population. Finally, the samples used in this study are just from the United States more than 20 years ago (NHANES 1999–2000) because the *H. pylori* serological data were only available in this period. Although these samples may not reflect the current socio-economic factors, we believe they still possess intrinsic value for research purposes.

## 5 Conclusion

In summary, this analysis of the general US population revealed an inverse association between PIR values and both *H. pylori* seropositivity and all-cause mortality. While *H. pylori* seropositivity is associated with all-cause mortality, it is not a major risk factor for all-cause mortality and does not appear to influence the relationship between PIR and all-cause mortality. These findings suggest that there is a need to strengthen screening for *H. pylori* infection, particularly among impoverished populations, and to enhance healthcare services to reduce *H. pylori* infection rates and mortality. Furthermore, our study emphasizes the value of assessing PIR values in patient populations to better inform public health strategies.

## Data Availability

Publicly available datasets were analyzed in this study. This data can be found here: https://www.cdc.gov/nchs/nhanes.

## Footnotes

^1^ https://www.cdc.gov/nchs/data-linkage/mortality.htm
