# Supplementary material for: Evaluating the relationship between familial poverty, Helicobacter pylori seropositivity, and all-cause mortality in the general US population
Source: Front Public Health. 2025 Jun 17;13:1578257. doi: 10.3389/fpubh.2025.1578257 (PMC12209254; doi:10.3389/fpubh.2025.1578257)
Supplement: Supplementary file 1 [file Data_Sheet_1.docx]

# Supplementary Figures


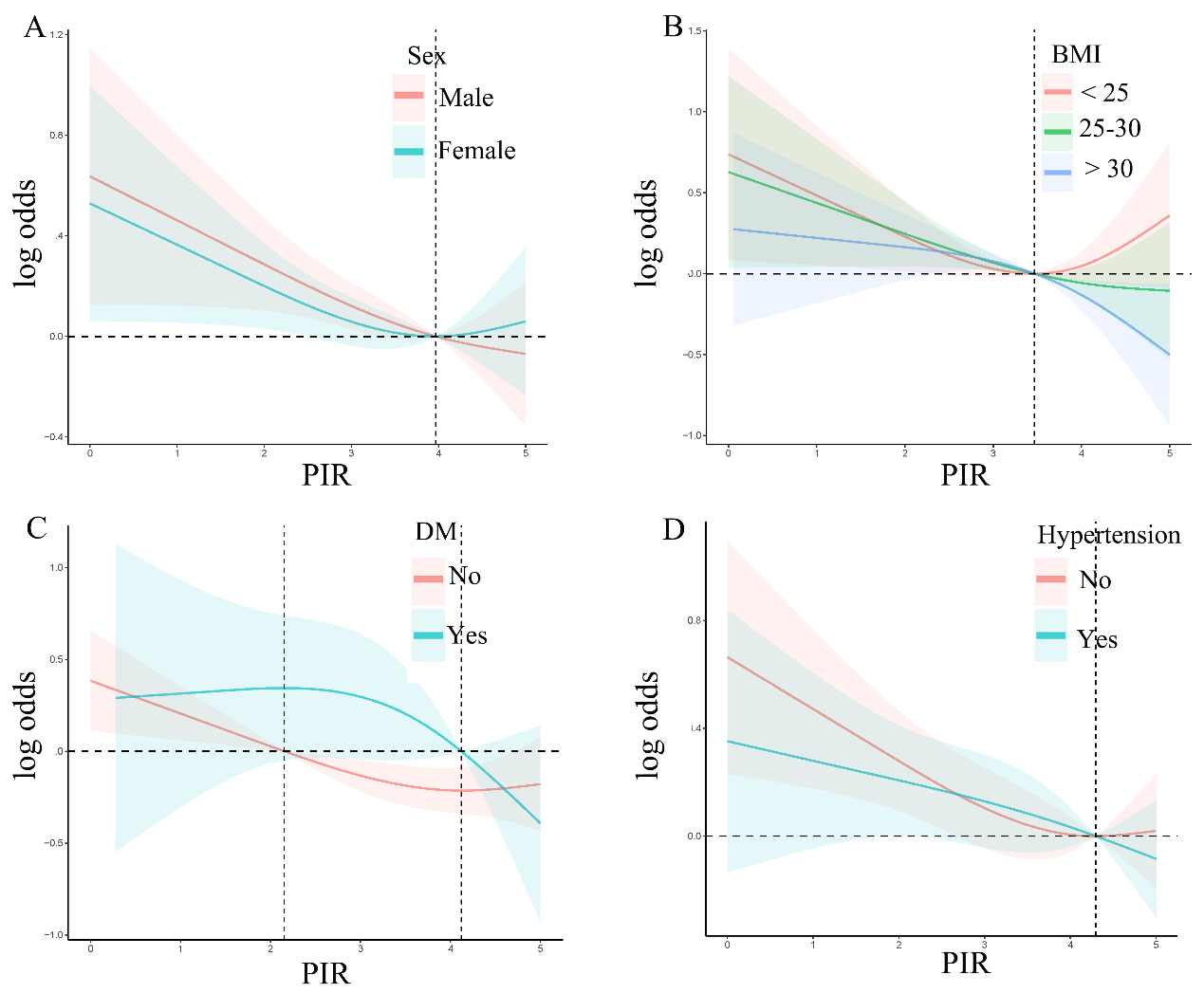


Supplementary Figure 1: The relationship between PIR values and *H. pylori* status stratified according to gender (A, orange and green shaded areas represent the confidence intervals for male and female), BMI (B, orange, green and blue shaded areas represent the confidence intervals for BMI < 25, 25-30, and >30), diabetes mellitus (C, orange and green shaded areas represent the confidence intervals for DM and without DM), and hypertension (D, orange and green shaded areas represent the confidence intervals for hypertension and without hypertension).

# Supplementary Tables

Supplementary Table 1: Sample-weighted subgroup analyses of the association between poverty-to-income ratio values and all-cause mortality.

|  | **HR** | **95% CI** | ***P* value** | ***P* for interaction** |
| --- | --- | --- | --- | --- |
| **Sex** |  |  |  | 0.26 |
| Female | 0.8 | 0.75-0.86 | <0.0001 |  |
| Male | 0.85 | 0.79-0.91 | <0.0001 |  |
| **Race/ethnicity** |  |  |  | 0.26 |
| Mexican American | 1.01 | 0.78-1.31 | 0.93 |  |
| Non-Hispanic Black | 0.85 | 0.77-0.95 | 0.003 |  |
| Non-Hispanic White | 0.78 | 0.72-0.83 | <0.0001 |  |
| Other Hispanic | 0.69 | 0.50-0.95 | 0.02 |  |
| Other Race/ethnicity | 0.82 | 0.65-1.04 | 0.1 |  |
| **Educational status** |  |  |  | 0.84 |
| Less than high school | 0.96 | 0.80-1.14 | 0.63 |  |
| High school or equivalent | 0.89 | 0.80-0.99 | 0.03 |  |
| College or above | 0.89 | 0.81-0.98 | 0.02 |  |
| **BMI (kg.m2)** |  |  |  | 0.37 |
| <25 | 0.85 | 0.78-0.94 | <0.001 |  |
| 25-30 | 0.79 | 0.73-0.86 | <0.0001 |  |
| >30 | 0.84 | 0.77-0.93 | <0.001 |  |
| **DM** |  |  |  | 0.73 |
| No | 0.85 | 0.80-0.89 | <0.0001 |  |
| Yes | 0.83 | 0.75-0.92 | <0.001 |  |
| **Hypertension** |  |  |  | 0.17 |
| No | 0.86 | 0.79-0.93 | <0.001 |  |
| Yes | 0.81 | 0.77-0.86 | <0.0001 |  |
| **Coffee intake** |  |  |  | < 0.0001 |
| No | 0.9 | 0.84-0.96 | <0.001 |  |
| Yes | 0.75 | 0.70-0.81 | <0.0001 |  |
| **Smoke** |  |  |  | 0.002 |
| Never | 0.79 | 0.74-0.84 | <0.0001 |  |
| Former | 0.75 | 0.68-0.83 | <0.0001 |  |
| Now | 0.97 | 0.87-1.08 | 0.6 |  |
| **Alcohol drinking** |  |  |  | 0.11 |
| Never | 0.88 | 0.80-0.97 | 0.01 |  |
| Former | 0.77 | 0.69-0.85 | <0.0001 |  |
| Moderate | 0.84 | 0.73-0.95 | 0.01 |  |
| Mild | 0.83 | 0.76-0.90 | <0.0001 |  |
| Heavy | 0.98 | 0.80-1.21 | 0.85 |  |

BMI, body mass index; DM, diabetes mellitus.

Supplementary Table 2: Sample-weighted subgroup analyses of the association between *H. pylori* serostatus and all-cause mortality.

|  | **HR** | **95% CI** | ***P* value** | ***P* for interaction** |
| --- | --- | --- | --- | --- |
| **Sex** |  |  |  | < 0.001 |
| Female | 1.4 | 1.26-1.55 | <0.0001 |  |
| Male | 1.16 | 1.03-1.30 | 0.01 |  |
| **Race/ethnicity** |  |  |  | 0.12 |
| Mexican American | 1.19 | 0.96-1.46 | 0.11 |  |
| Non-Hispanic Black | 1.35 | 1.25-1.47 | <0.0001 |  |
| Non-Hispanic White | 1.42 | 1.27-1.57 | <0.0001 |  |
| Other Hispanic | 1.03 | 0.76-1.38 | 0.86 |  |
| Other Race/ethnicity | 1.24 | 0.90-1.71 | 0.19 |  |
| **Educational status** |  |  |  | 0.16 |
| Less than high school | 1.12 | 0.96-1.31 | 0.14 |  |
| High school or equivalent | 1.1 | 0.94-1.29 | 0.25 |  |
| College or above | 1.27 | 1.12-1.45 | <0.001 |  |
| **BMI (kg.m2)** |  |  |  | 0.36 |
| <25 | 1.19 | 1.02-1.40 | 0.03 |  |
| 25-30 | 1.24 | 1.10-1.40 | <0.001 |  |
| >30 | 1.35 | 1.18-1.54 | <0.0001 |  |
| **DM** |  |  |  | 0.76 |
| No | 1.23 | 1.11-1.36 | <0.001 |  |
| Yes | 1.2 | 1.01-1.42 | 0.04 |  |
| **Hypertension** |  |  |  | 0.56 |
| No | 1.27 | 1.07-1.49 | 0.01 |  |
| Yes | 1.21 | 1.11-1.31 | <0.0001 |  |
| **Coffee intake** |  |  |  | 0.27 |
| No | 1.31 | 1.13-1.52 | <0.001 |  |
| Yes | 1.21 | 1.11-1.32 | <0.0001 |  |
| **Smoke** |  |  |  | 0.21 |
| Never | 1.34 | 1.20-1.50 | <0.0001 |  |
| Former | 1.2 | 1.06-1.36 | 0.003 |  |
| Now | 1.19 | 1.03-1.39 | 0.02 |  |
| **Alcohol drinking** |  |  |  | 0.45 |
| Never | 1.14 | 0.93-1.40 | 0.22 |  |
| Former | 1.32 | 1.20-1.44 | <0.0001 |  |
| Moderate | 1.31 | 1.07-1.61 | 0.01 |  |
| Mild | 1.25 | 1.14-1.37 | <0.0001 |  |
| Heavy | 1.09 | 0.80-1.47 | 0.59 |  |

BMI, body mass index; DM, diabetes mellitus.
